# Emerging Themes and Research Frontiers in Suicide Prediction: A Scientometric Analysis

**DOI:** 10.7759/cureus.62139

**Published:** 2024-06-11

**Authors:** Kochumol Abraham, Anish K R, Greety Toms, Nice Mary Francis P, Jobi Babu

**Affiliations:** 1 Department of Computer Applications, Marian College Kuttikkanam, Peermade, IND; 2 Department of Social Work, Rajagiri College of Social Sciences, Kalamassery, IND; 3 Department of Library and Information Sciences, Bharta Mata College, Thrikkakara, IND; 4 Department of Psychology, Prajyoti Niketan College, Thrissur, IND; 5 Department of Social Work, Marian College Kuttikkanam, Peermade, IND

**Keywords:** vosviewer, biblioshiny, bibliometric analysis, scientometric analysis, suicide prediction

## Abstract

Suicide remains a critical global health issue despite advancements in mental health treatment. The purpose of this analysis is to emphasize the development, patterns, and noteworthy outcomes of suicide prediction research. It also helps to uncover gaps and areas of under-researched topics within suicide prediction. A scientometric analysis was conducted using Biblioshiny and VOSviewer. To thoroughly assess the academic literature on suicide prediction, various scientometric methodologies such as trend analysis and citation analysis were employed. We utilized the temporal features of the Web of Science to analyze publication trends over time. Author affiliation data were used to investigate the geographic distribution of research. Cluster analysis was performed by grouping related keywords into clusters to identify overarching themes within the literature. A total of 1,703 articles from 828 different sources, spanning from 1942 to 2023, were collected for the analysis. Machine learning techniques might have a big influence on suicide-related event prediction, which would enhance attempts at suicide prevention and intervention. The conceptual understanding of suicide prediction is enhanced by scientometric analysis, which further uncovers the research gap and literature in this area. Suicide prediction research underscores that suicidal behavior is not caused by a single factor but is the result of a complex interplay of multiple factors. These factors may include biological, psychological, social, and environmental factors. Understanding and integrating these factors into predictive models is a theoretical advancement in the field. Unlike previous bibliometric studies in the field of suicide prediction that have typically focused on specific subtopics or data sources, our analysis offers a comprehensive mapping of the entire landscape. We encompass a wide range of suicide prediction literature, including research from medical, psychological, and social science domains, thus providing a holistic overview.

## Introduction and background

The World Health Organization (WHO) emphasizes suicide prevention as a global imperative [1]. It suggests that risk, protective factors, and related interventions should be systematically taken into account to prevent suicide [1]. WHO specifically recommends that indicated methods protect people at risk, universal strategies target whole populations, and selected initiatives target higher-risk groups [1]. Evidence suggests that restricting access to means is the most effective prevention method, surpassing high-risk strategies aimed at individuals with a history of suicidal behavior. The best evidence for prevention comes from limiting the availability of methods. Compared to high-risk techniques that target patients who have considered or attempted suicide, population-based initiatives are more successful [2,3]. Advances in technology, particularly machine learning, offer the potential for predicting suicide risk by analyzing social media data to identify long-term contextual factors associated with suicidal thoughts and actions [4-6]. Durkheim’s foundational work and Bronfenbrenner’s ecological systems theory have informed the categorization of suicide risk factors [7-9]. The study of how suicidal thinking develops into suicidal conduct has also drawn more interest in recent years. The existence of moderators, such as having access to suicide tools, being more capable of attempting suicide, being exposed to suicide, and experiencing impulsive sentiments, facilitates the shift from suicidal ideation to behavior [10].

The scientometric analysis is used to map the research structure and evaluate the factual production quantitatively [11-14]. Analyzing publishing, citation, and cooperation patterns within an area of study or among scholars in general is the goal of this method [15-17]. Connecting articles, journals, authors, keywords, and co-occurrence networks allows for many interpretations and can help shape future trends and directions [18,19]. Measuring the influence of research publications and academic journals, comprehending scientific citations, and using these metrics in policy and management contexts are some of the major research challenges. The scientometric approach finds research subjects and creates a network model using bibliometric data [12,14]. To illustrate the intellectual perspective of a certain knowledge domain, scientometric analysis creates network models, which can help researchers achieve their study goals and find answers to their problems.

One of the key R programs in the R-studio (version 4.2.1) is Bibliometrix, which is used for scientometric research [20-23]. We were able to obtain the publications’ essential and fundamental data by extracting raw data from the Web of Science and importing it into the Biblioshiny website [24,25]. This data included the publications’ period, number of documents, number of sources, authors, authors’ collaboration, number of references, document content, and document types. With the data, we were able to quickly determine if the outcomes met our expectations. Additional data included the annual production of scientific research, the average number of references per paper, and the most cited documents and authors [26,27]. VOSviewer, a visualization tool developed by Leiden University, is used for network visualization [28-30].

Utilizing bibliometric and scientometric analysis, this study seeks to address the following research questions: RQ1: Which are the factors that highly contribute to suicide prediction? RQ2: What are the key trends in suicide prediction research? RQ3: Which authors and journals are the most prolific in suicide prediction metrics? RQ4: What are the future trends of publications on suicide prediction studies?

## Review

Literature review

The increasing prevalence of suicide worldwide is alarming and warrants the attention of researchers and clinicians. Over the years, conventional methods of suicide prediction have been seen as suboptimal, prompting a shift toward more advanced technological approaches such as artificial intelligence (AI) in predicting suicide risks. The presented literature reviews various AI and machine learning models used in predicting suicidal tendencies based on online social media interactions and other behavioral cues. MacKinnon et al. (1976) assessed the utility of suicide prediction. Their analysis revealed the difficulty in predicting suicide beyond a 20% level of efficiency, challenging the assumption that most suicides can be reliably predicted [31]. Balon et al. (1987) discussed the challenges of predicting suicide, emphasizing that suicide cannot be reliably predicted. This historical perspective highlights the ongoing complexity of suicide prediction [32]. Davis et al. (1990) reviewed sociodemographic and psychiatric predictors of suicide risk, highlighting the challenges of predicting suicide. They called for more research into the factors that distinguish suicidal individuals from non-suicidal individuals and emphasized the need for therapeutic relationships [2]. Eyman et al. (1991) assessed the impact of personality tests on suicide prediction accuracy. Their examination clarified the psychological aspects of forecasting [33]. Coryell et al. (2007) examined biological markers for the prognosis of suicide, focusing particularly on anomalies in serotonin modulation and hypothalamic-pituitary-adrenal axis functioning. Their work showed that serum cholesterol concentrations combined with the results of a dexamethasone suppression test can produce clinically relevant estimates of suicide risk, underscoring the potential use of biological testing in prediction models [34]. Stefansson et al. (2012) assessed the predictive validity of the Suicide Intent Scale (SIS) in patients at a high risk of suicide. The study found that SIS is a useful technique for clinical suicide risk assessment, especially when applied in a simplified manner [35]. Delgado-Gomez et al. (2012) based on personality features and life events used multivariate algorithms to classify suicide attempters with a reasonable degree of accuracy. This illustrates the use of multivariate approaches in suicide prediction [36]. Hawgood et al. (2016) highlighted the need for a paradigm change in the analysis of suicide risk. They contended that to increase precision and efficacy in identifying those at risk, it may be necessary to reevaluate present techniques [37]. Clive et al. (2016) introduced a DNA methylation biosignature to enhance suicide prediction models, exploring the overlap between stress-related states and suicide-associated DNA methylation for improved prediction [38]. Their research explored the overlap between stress-related states and suicide-associated DNA methylation, potentially providing a biological basis for improved prediction [38]. D’Hotman et al. (2020) delved into the potential of AI in suicide prediction. They conducted a qualitative narrative review, highlighting two core areas, namely, medical suicide prediction and social suicide prediction. The initial findings indicated that AI might enhance our capacity to detect those vulnerable to suicide. However, they recommended further research to validate these tools across diverse contexts, emphasizing the need for independent oversight, especially for private entities such as Facebook [39]. Corke et al. (2020) conducted a meta-analysis of exploratory suicide prediction models, ranging from those based on clinical judgment to machine learning approaches. Their analysis of numerous studies revealed that machine learning models may offer a higher odds ratio in suicide prediction compared to clinical judgment. Furthermore, models incorporating a larger number of suicide risk factors tend to perform better, particularly in machine learning studies [40]. According to Rezig et al. (2021), with the surge in social media usage, platforms such as Twitter have become potential grounds for identifying suicidal tendencies. Rezig et al. employed a machine learning model optimized with GW Life GWO to detect suicidal ideation in Twitter users. The focus was on changing the emotional states of users. The findings emphasized the model’s efficiency in pinpointing suicidal tendencies, showing promising recall and precision results [41]. Kumar et al. (2021) introduced a novel methodology combining social and topic context for sentiment analysis on microblogs, such as tweets. They incorporated the notion of structure similarity in social contexts and combined it with topic context. Experimental results showed that their model outperformed baseline methods, proving beneficial for suicide prediction [42]. Menon et al. (2023) discussed the potential of AI-based models for suicide prediction. They highlighted that conventional approaches have limitations and suggest that AI-driven methods hold promise, especially in low- and middle-income countries. This study emphasizes the evolving role of AI in suicide prediction [43].

Methodology

The core collection of the Web of Science database provided us access to the scientific papers pertinent to the study. We conducted a search on October 11, 2023, using certain keywords such as “suicide” and “suicide prediction.” The data comprised conference papers, reviews, and articles from peer-reviewed journals. The search was performed without applying language restrictions. We retrieved 1,583 articles between 1942 and 2023 from 828 distinct sources. We eliminated any duplicates from the Web of Science core collection to guarantee correctness and 1,328 were selected for the study. The outcomes were stored as a “csv” file, and we used VOSviewer version 1.6.19 and Biblioshiny software to perform a bibliometric analysis of the data. Table 1 includes comprehensive details about the main elements and facets of the research.

**Table 1:** Critical aspects of the investigation.

Description	Results
Main information about the data	
Timespan	1990–2023
Sources (journals, books, etc)	464
Documents	1,328
Annual growth rate (%)	10.74
Document average age	9.1
Average citations per document	39.88
References	41,453
Document content	
Keywords Plus (ID)	2,639
Author’s keywords (DE)	2,270
Authors	
Authors	5,860
Authors of single-authored docs	66
Authors’ collaboration	
Single-authored documents	74
Co-authors per document	5.83
International co-authorships (%)	22.97
Document types	
Article	1,280
Article, book chapter	1
Article, early access	27
Article, proceedings paper	19
Correction, early access	1

Results

Annual Scientific Production

**Figure 1:**
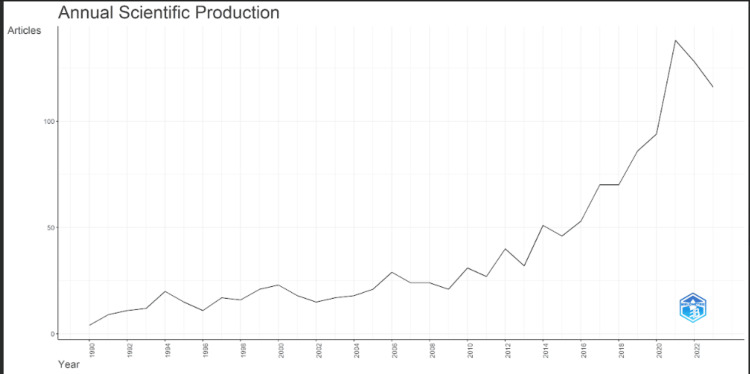
Annual scientific production from 1990 to 2023.

Between 1990 and 2023, the number of publications related to suicide prediction increased and then decreased. Still, there was a significant upsurge in published works between 2015 and 2021, which was followed by a downturn. Over the years, there has been a clear cyclical pattern of alternating gains and reductions. Biblioshiny was used to construct the visual representation shown in Figure 1, which illustrates the relationship between the number of publications and the years they were published.

Most Relevant Sources

**Figure 2:**
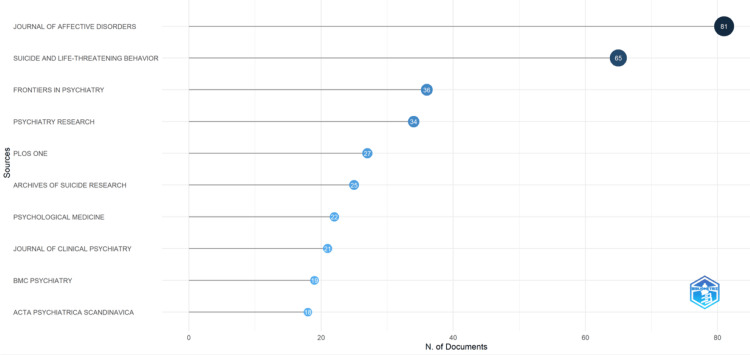
Top 10 relevant sources.

From the 1,328 collected publications, the Journal of Affective Disorders stood out as the most productive journal analyzed, with a maximum of 81 articles. The journal Suicide and Life-Threatening Behavior followed closely behind with 65 publications. Figure 2 lists the top 10 journals that produced the most suicide prediction analysis papers. It helps us understand which journals are the most prolific or have published the most articles in this field.

Historiographic Analysis

**Figure 3:**
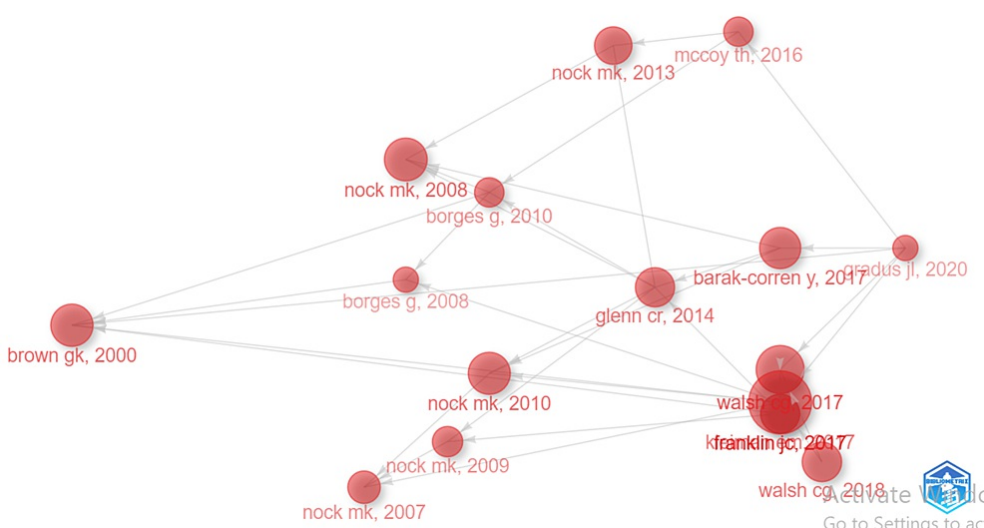
Chronological map of the most relevant citations.

Figure 3 shows a chronological network map of the most relevant direct citations found in a bibliographic collection. It reflects the growing importance and diversification of suicide-related research from 2000 to 2020. All papers in this network are assigned to cluster 1. This suggests that they are part of the common theme “suicide prediction” and are closely related in terms of this theme. The Local Citation Score (LCS) is a measure of how often the paper has been cited within a specific field or local academic community. It reflects the paper’s impact within a particular academic niche, whereas the Global Citation Score (GCS) is a measure of how often the paper has been cited globally across various academic disciplines and communities. It reflects the paper’s broader impact and influence in the academic world. The paper entitled “Risk Factors for Suicidal Thoughts and Behaviors: A Meta-Analysis of 50 Years of Research” published in Psychological Bulletin by Franklin JC is the most significant and highly influential paper in the area of suicide prediction as it is the most locally and globally cited paper with a score of 180 for LCS and 1,768 for GCS. The high citation impact of this paper underscores its significance in shaping the field of suicide research and policy. Researchers and policymakers in this field might use this dataset to identify relevant papers, gather insights, or track the evolution of research in this area.

Trend Topics

**Figure 4:**
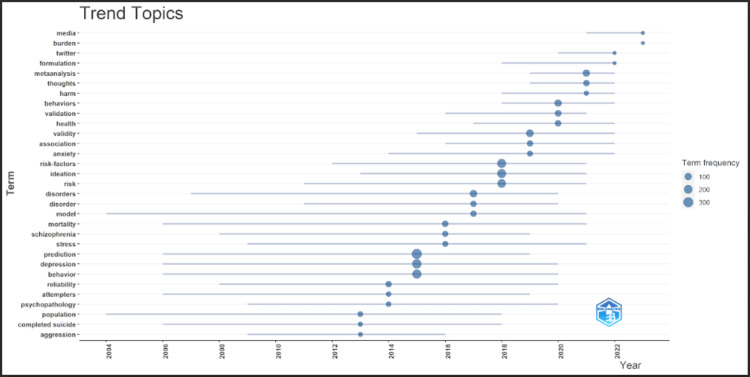
Representation of trend topic.

Figure 4 depicts a hierarchical representation of various keywords in suicide prediction. These topics are considered the core topics in discussion by research scholars from 2013 to 2022. The most discussed topic in 2013 was population. Similarly, in 2014, reliability was the leading topic. In 2015, prediction was the most trending. However, in 2020, behavior was top on the list, whereas in 2023, media was the trending topic.

Thematic Focus on Suicide Prediction

**Figure 5:**
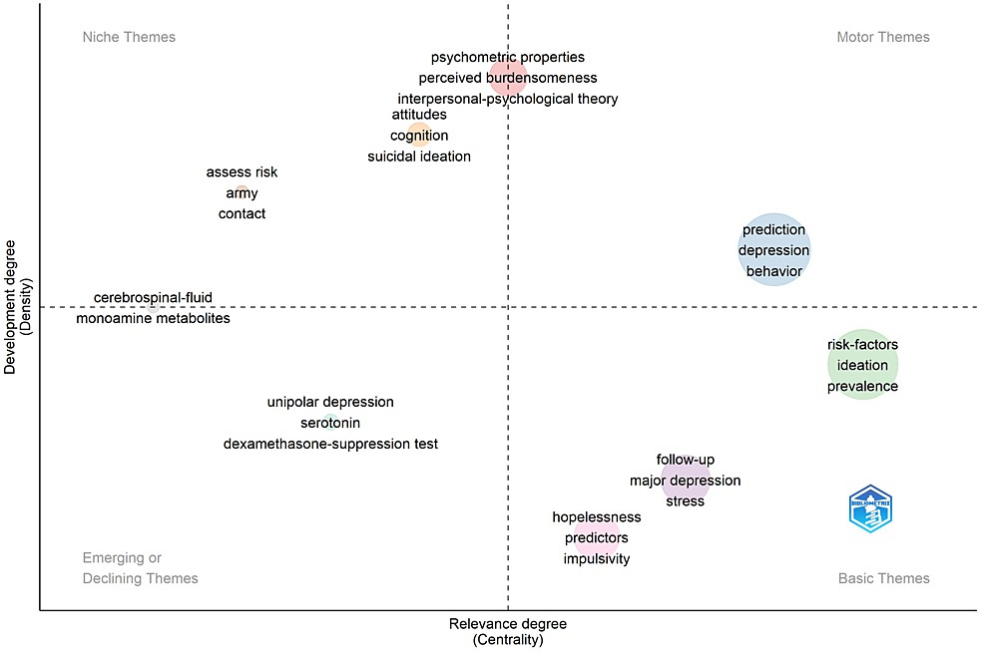
Thematic representation of suicide prediction.

This section analyzes the key trends in suicide prediction research and highlights the focus areas that have garnered researchers’ attention over time. A thematic map is an instinctive plot that enables us to examine themes in terms of the quadrants in which they are positioned. An analysis of the subject’s present situation and potential for sustenance is the goal of a thematic map. The results of this investigation help educate scholars about the possibility of the establishment of new thematic research topics within a discipline. The authors’ keyword clusters and their associations are examined in the thematic analysis to derive themes. The two properties of centrality and density are commonly used to define these topics. The vertical axis indicates density and the horizontal axis indicates centrality. While centrality describes the level of association between various subjects, density measures the cohesion among the nodes. The relevance and degree of advancement of a topic are determined by these two elements. Centrality and relevance are based on the number of connections a node has with other nodes, placing it in a crucial spot. Similarly, the bonding between the nodes that make up a research field’s density determines its ability to expand and support itself. According to Callon’s centrality and density rank, the co-occurrence network clusters are displayed as bubbles in the thematic mapping analysis [44]. The number of words in a cluster is indicated by the bubble size. The relevance of a theme is measured along the X-axis, which shows the degree of interaction with other graph clusters. The cluster network’s internal strength, theme growth, and density are all shown on the Y-axis [45-47]. The thematic map for the area of suicide prediction is shown in Figure 5. There are four quadrants on the map, numbered Q1 through Q4. The upper right quadrant (Q1) represents “motor themes” that drive the field, the lower right quadrant (Q4) represents “basic themes” that provide foundational knowledge, the upper left quadrant (Q2) contains “niche themes” that are highly specialized, and the lower left quadrant (Q3) includes “emerging or declining themes” that are either gaining or losing relevance.

Interpretation of Thematic Map

Themes such as “cerebrospinal fluid” and “monoamine metabolites,” jammed between Q1 and Q4, are well-developed and competent in the research domain. In other words, “cerebrospinal fluid” and “monoamine metabolites” remain the leading themes within the field.

Themes in Q2 are getting internally harmonious but have only made a little impact on the area of suicide prediction. The information indicates that themes from Q1 such as prediction, depression, and behavior ought to be more closely related to suicide prediction.

The themes in Q3 such as “unipolar depression,” “serotonin,” and “dexamethasone-depression tests” appear to be the emerging themes.

Themes in Q1 such as “psychometric properties,” “perceived burdensomeness,” and “interpersonal psychological theory” appear to be driving themes, even then they appear to be niche topics as they traverse to Q2.

Factorial Analysis

Factor analysis by keywords is a powerful technique in bibliometric analysis, allowing researchers, institutions, and policymakers to gain insights into the structure and dynamics of research areas and trends based on the terms used in academic publications. Table 2 depicts a factor analysis using keywords in the area of suicide prediction.

**Table 2:** Factorial analysis using keywords.

Word	Dim.1	Dim.2	Cluster
Prediction	-0.36	-0.08	1
Depression	0.21	0.67	1
Behavior	-0.06	0.64	1
Risk factors	0.53	-0.09	1
Ideation	0.92	0.63	1
Risk	-0.31	0.06	1
Prevalence	0.35	0.18	1
Suicide	-0.96	-0.19	1
Validity	0.89	0.51	1
Disorders	-0.15	-0.08	1

The values labeled “Dim.1” and “Dim.2” represent the positions of each term in a reduced two-dimensional space. An interpretation of the table is provided below.

Cluster: All entries in the table are grouped together in Cluster 1. This indicates that they have similar traits or are closely connected to each other within the reduced space represented by “Dim.1” and “Dim.2.” Dim.1 (Dimension 1): Dim.1 represents principle component 1 or the primary theme of the data. An interpretation of the terms in cluster1 positioned along Dim.1 is presented below.

“Prediction,” “behavior,” and “risk” have negative values in “Dim.1.” It indicates that they are inversely related to the primary theme. “Depression,” “risk factors,” “ideation,” “prevalence,” and “validity” have positive values which suggest that they are closely associated with the primary theme captured by “Dim.1.”

“Suicide” and “disorders” have strong negative values in “Dim.1,” which suggests that they are distinctly unrelated to the primary component or may even be inversely related to it.

Dim.2 (Dimension 2) represents principle component 2 or the secondary theme of the data. An interpretation of the terms in Cluster1 positioned along Dim.2 is presented below.

“Prediction,” “risk factors,” “suicide,” and “disorders” have negative values in “Dim.2,” which indicates that they are inversely related to principle component 2 or the secondary theme. “Depression,” “behavior,” “ideation,” “risk,” “prevalence,” and “validity” have positive values which suggest that they are closely associated with principle component 2 captured by “Dim.1.”

Document Citation Analysis

**Figure 6:**
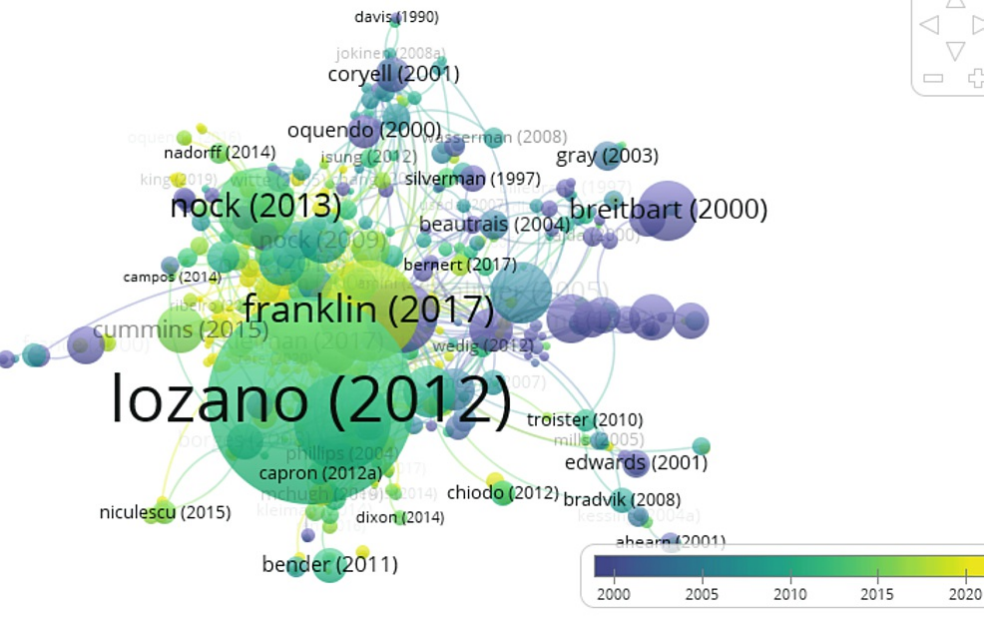
Visual representation of the citation relationships between documents.

The citation linkages between documents are shown visually in a document citation analysis chart. It aids in the identification of important documents, the comprehension of citation trends, and the identification of connected research clusters. Finding significant works in a subject of study and performing bibliometric analysis can benefit from this. It also aids in learning about the progression of an author's research interests, partnerships, and publishing history over time. Finding an author’s key works throughout several years, monitoring research trends, and comprehending the dynamics of an author’s scholarly output over time can all benefit from it. The fact that the author’s name and the year of publication are included in the same node implies that each node comprises an author together with one or more documents that they have written within a specific year. VOSviewer Version 1.6.19 was used for this analysis. Here, we took the minimum number of citations as 10. An overlay visualization of the 731 documents, with the minimum number of citations as 10, is shown in Figure 6.

Co-occurrence of Keywords

**Figure 7:**
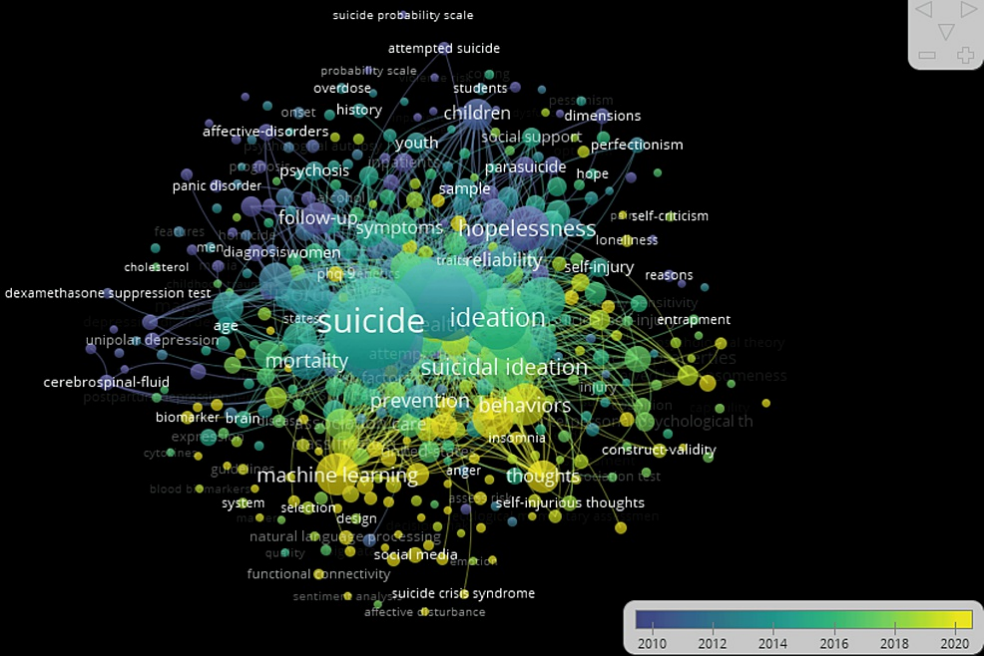
Co-occurrence clusters in suicide prediction.

A visualization of term co-occurrence clusters in suicide prediction was done using the VOSviewer software. For co-occurrence analysis, a subset of 427 keywords, i.e., keywords that occurred five times or more was chosen. Figure 7 presents the findings. The size of the nodes and font depends on each keyword’s weight value. The weight value indicates how frequently a keyword appears, with larger nodes and fonts corresponding to keywords that appear more frequently. The lines connecting the nodes represent common occurrences between keywords. The thickness of these lines indicates the strength of co-occurrence between the two keywords. A thicker line signifies a higher frequency of co-occurrence. Based on the analysis, nine distinct clusters were identified. The first cluster comprises 79 items, the second consists of 69 items, the third comprises 57 items, the fourth comprises 56 items, the fifth comprises 52 items, the sixth comprises 44 items, the seventh comprises 27 items, the eighth comprises 26 items, and the ninth consists of 17 items.

Bibliographic Coupling

**Figure 8:**
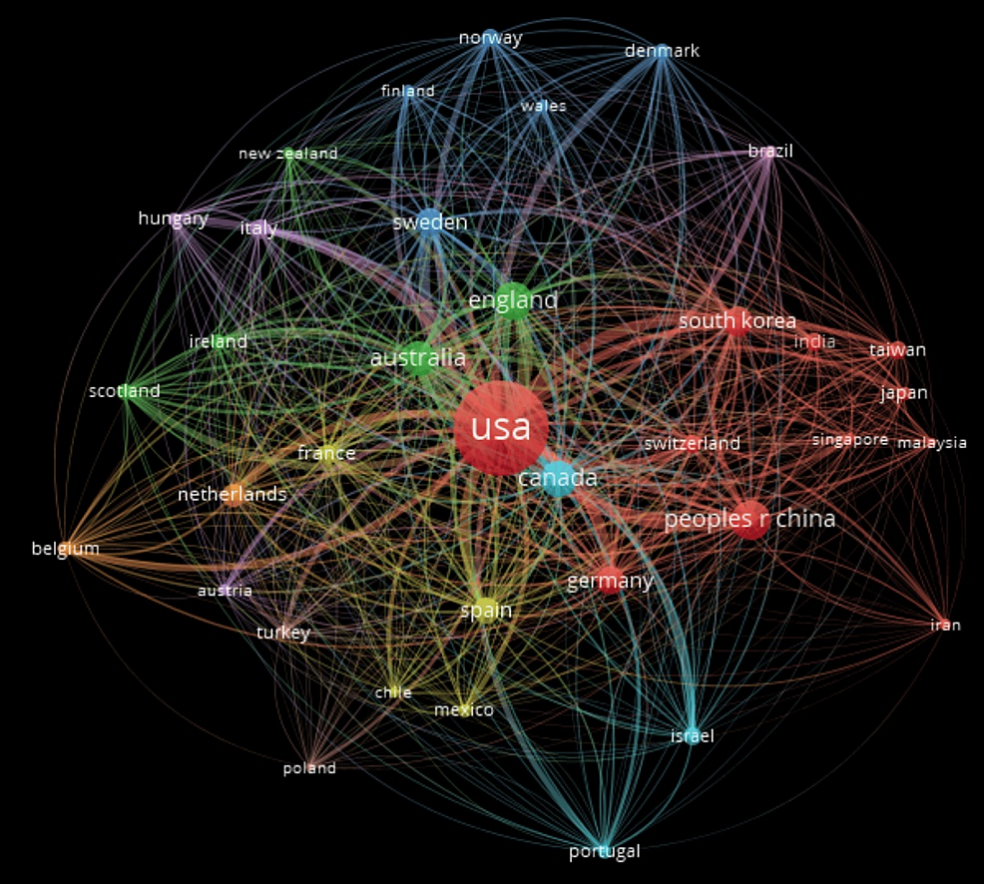
Network representation of bibliographic linkage among 39 nations.

Country-wise bibliographic coupling is a bibliometric analysis method that involves examining the connections between scientific or scholarly articles based on the common references they cite, with a specific focus on the countries of origin of the publications. It can provide insights into the collaboration and research trends of different countries. Here, we analyzed the connections between articles based on the common references they cite. In a country-wise bibliographic understanding, two articles from separate nations are deemed to be coupled when they have shared references. A network visualization produced using VOSviewer is shown in Figure 8. As shown in the figure, the United States is the most significant country in suicide prediction research.

Discussion

Bibliometric analysis can be used to determine the population demographics at risk for suicide as well as the risk factors for suicide. Strategies for prevention and focused treatments can be created with this information. Additionally, it can be used to determine which suicide prediction strategies indicate the greatest possibility. Both new and improved suicide prediction models can be created using this data, as well as current ones. It can be used to highlight areas that require more investigation as well as gaps in the body of knowledge regarding suicide prediction. Using this data, suicide research can be conducted more effectively and efficiently, and the creation of successful suicide prevention plans can proceed more efficiently.

Researchers and information scientists might learn more about the emergence and progression of research trends by examining bibliographic coupling. Based on how many articles pair with a specific set of references, they may monitor how a topic becomes more or less popular over time. Funding agencies and policymakers can gain insights from a country-by-country bibliographic coupling study. They can use the findings to pinpoint areas that would benefit from international cooperation or funding.

By counting the number of articles that pair with a specific set of references, they may monitor the rise or fall in popularity of a topic over time. For funding organizations and policymakers, a country-wise bibliographic coupling analysis can yield useful information. They can use the findings to determine areas that should get investment or where international collaboration can be promoted. Factor analysis can help reduce the dimensionality of the data and identify underlying structures, which can be valuable in various fields such as psychology, social sciences, marketing, and more. Thematic maps in suicide prediction are valuable tools for visualizing and understanding the spatial aspects of suicide risk. They can aid policymakers, mental health professionals, and researchers in making informed decisions, directing resources effectively, and developing targeted interventions to prevent suicide. Historiographical analysis helps researchers and practitioners in suicidology gain a deeper understanding of the field’s historical foundations, challenges, and successes. It can inform the development of more effective suicide prediction models and prevention strategies by drawing on the lessons learned from the past. Additionally, it can contribute to a more ethical and nuanced approach to suicide prediction in contemporary society. Here, we can explore how research trends develop over time by examining the most-cited papers and their influence on subsequent research. Additionally, we can look for emerging topics and key publications that have catalyzed new areas of study.

Recommendations

The integration of machine learning and AI mechanisms into suicide prediction holds great promise for improving mental healthcare. However, responsible development, ethical considerations, and ongoing research are essential to maximize the benefits while minimizing potential risks. Collaborative efforts across multiple domains are crucial to advance the field and ultimately save lives. We recommend the establishment of repositories that curate de-identified mental health datasets for researchers, ensuring data privacy and ethical considerations. The research on suicide prediction is a complex and multidisciplinary field. Fostering interdisciplinary collaborations among researchers, clinicians, data scientists, and experts in machine learning and AI is highly recommended. Such collaborations can lead to a holistic approach to suicide prevention. The design and implementation of AI-driven suicide prediction tools should be user-centered. Predicting and preventing suicide will be aided by utilizing machine learning techniques to find new indications and forecast variables. Natural language processing techniques can analyze text data, such as social media posts, clinical notes, or suicide notes, to identify linguistic cues associated with suicide risk. Time-series analysis techniques can be used to monitor behavioral changes over time, which may reveal variations in an individual’s mental health. Predictive accuracy can be increased by combining many models using ensemble approaches such as gradient boosting. Sentiment analysis is a useful tool for evaluating the emotional content of textual material and identifying indications of suffering in spoken or written language. Instead of being viewed as a substitute for clinical assessment, machine learning can be used as an additional tool to help mental health providers and caregivers. The accuracy and reliability of bibliometric studies heavily depend on the quality of the underlying data. In suicide prediction research, this can be particularly challenging due to the sensitive nature of the topic and potential biases in reporting. There may be a bias toward publishing positive results or studies with statistically significant findings, which could skew the bibliometric analysis toward certain types of research and potentially obscure important negative or null results.

## Conclusions

Our scientometric analysis has shed light on the current status of suicide prediction research. We gained a better grasp of the present suicide prediction landscape by identifying important research works, uncovering important patterns, and thoroughly analyzing the body of existing literature. Researchers and information scientists might learn more about the emergence and progression of research trends by examining bibliographic coupling. Based on how many articles pair with a specific set of references, they may monitor how a topic becomes more or less popular over time. According to our data, the pressing need for successful suicide prevention techniques has led to a rise in interest in suicide prediction research over the past few decades. The literature shows a move away from conventional risk factor-based strategies and toward more sophisticated, interdisciplinary approaches that use machine learning, AI, and advanced data analytics. In summary, it provides a thorough review of suicide prediction studies and lays the groundwork for more investigation and creativity in this important field. Bibliometric analyses often rely on databases that primarily index publications in English, which could lead to a language bias and potentially exclude relevant research published in other languages. It takes time for research findings to be published, indexed, and included in bibliometric databases. As a result, bibliometric studies may not capture the most recent developments in suicide prediction research, leading to a time lag in the analysis. We may create techniques that are more sensitive, accurate, and successful in detecting people who are at risk of suicide and, eventually, in saving lives if we use the insights and lessons gained from this investigation.
